# Approaching High-Performance TS-1 Zeolites in the Presence of Alkali Metal Ions via Combination of Adjusting pH Value and Modulating Crystal Size

**DOI:** 10.3390/nano13162296

**Published:** 2023-08-10

**Authors:** Geng Li, Kairui Fu, Fulin Xu, Tianduo Li, Yunan Wang, Jingui Wang

**Affiliations:** 1 Shandong Provincial Key Laboratory of Fine Chemicals, School of Chemistry and Chemical Engineering, Qilu University of Technology (Shandong Academy of Sciences), Jinan 250353, China; iamligeng97@163.com (G.L.); kr475686761@163.com (K.F.); xufulin97@163.com (F.X.); ylpt6296@vip.163.com (T.L.); 2 School of Chemistry and Chemical Engineering, University of Jinan, Jinan 250022, China; 3 Key Laboratory of Advanced Fuel Cells and Electrolyzers Technology of Zhejiang Province, Ningbo Institute of Materials Technology and Engineering, Chinese Academy of Sciences, Ningbo 315201, China

**Keywords:** zeolites, TS-1, inorganic alkali, titanium, Lewis acid, oxidation

## Abstract

Lewis acid zeolites play an important role in industrially important green reactions closely related to fine chemical and biomass conversion. Titanium-doped TS-1 zeolite is a milestone Lewis acid zeolite widely used in industrially significant green oxidation processes with hydrogen peroxide as an oxidant under mild conditions. TS-1 zeolites are normally synthesized in basic conditions under hydrothermal treatment. Up to now, there has still been no success in synthesizing active TS-1 Lewis acid zeolites by using inorganic alkali, e.g., NaOH or KOH as base, which is cheaper and more stable compared to the quaternary ammonium hydroxide or organic amines used in traditional synthesis. Here, an inorganic base of NaOH was employed in synthesizing active TS-1 zeolites for the first time. The crucial factor was the control of adverse effects of sodium cations on the incorporation of active titanium cations. Higher catalytic activity was achieved by further reducing the size of the TS-1 crystal by using the seed-added strategy, which uses the catalytic activity of a commercial catalyst, the production cost being much lower than commercial TS-1 catalysts, indicating great commercial potential and the possibility of preparing other cheap Lewis acid catalysts by using inorganic alkali.

## 1. Introduction

Zeolites, a category of microporous crystalline solid catalysts, play important roles in petroleum refinement and fine chemical and biomass conversion [1,2,3,4,5,6,7,8,9,10,11]. Zeolites are usually synthesized in basic conditions under hydrothermal treatment [4,12,13,14]. Inorganic alkali, e.g., NaOH or KOH, is widely used to provide a basic condition during zeolite synthesis because it is a widely available and cheap basic source. Unfortunately, usage of these cheap inorganic bases is highly restricted in Lewis acid zeolite synthesis [15,16,17,18,19], especially for Ti-doped TS-1 zeolites [20,21,22,23,24,25,26,27,28], which are widely used in numerous industrially significant green oxidation processes with hydrogen peroxide as an oxidant under mild conditions [29,30,31,32,33,34,35,36]. The reason is that alkali metal cations, e.g., Na^+^, from inorganic alkali have an adverse effect on the incorporation of active Ti sites. The presence of alkali metal cations, even a trace amount of ~0.1 wt.% in the synthetic system, can lead to an absence of TS-1 catalytic activity [37,38]. Therefore, a quaternary ammonium hydroxide of tetrapropylammonium hydroxide (TPAOH) was chosen as both the organic structure-directing agent (TPA^+^) and basic source (OH^−^) in traditional synthesis for the first time. Due to the strict requirements of alkali metal cation content, high-purity TPAOH prepared using the AgOH and electrolytic methods is extremely expensive, leading to the high production cost of TS-1 zeolites in traditional synthesis. Thus, in order to reduce costs, various strategies have been designed by using tetrapropylammonium bromide (TPABr)—which has the same cation TPA^+^—with TPAOH as the structure-directing agent combined with ammonia or organic amines to provide a basic synthetic condition [39,40,41,42,43]. These methods significantly decrease production costs due to TPABr being much cheaper than TPAOH. However, the low alkalinity of ammonia and organic bases leads to increased usage amounts in the synthetic system. Moreover, the volatile properties of these bases creates environmental problems.

Considering the high alkalinity, low cost, and nonvolatile nature of NaOH, synthesizing TS-1 using NaOH as the base and combining it with cheap TPABr as a template is highly desirable. However, this is still very challenging due to the undesirable effects of Na^+^ during synthesis, as mentioned above. Therefore, if NaOH is chosen as the base for synthesis, the key to creating an active TS-1 zeolite is solving the adverse effect that Na^+^ has on active Ti sites.

Here, a novel strategy was developed to solve the problem of the adverse effects of Na^+^ ions via precisely controlling the pH value. Using an optimal recipe, the active TS-1 zeolites for 1-hexene epoxidation with H_2_O_2_ was obtained by using NaOH as the base and TPABr as the template. Higher catalytic activity was achieved by further reducing the size of the TS-1 crystal by using the seed-added strategy, which delivered similar catalytic performance to current commercial TS-1 catalysts that use TPAOH as a preparation template.

## 2. Materials and Methods

### 2.1. Materials

Titanium tetra-n-butoxide, colloidal silica, sodium hydroxide, tetrapropylammonium bromide, hydrogen peroxide, 1-hexene, cyclohexene, and cerium sulfate were bought from Macklin. All the chemical reagents were used without further purification.

### 2.2. Syntheses of TS-1 Catalysts

The colloidal silica and titanium tetra-n-butoxide were employed as silicon and titanium sources, respectively. Tetrapropylammonium bromide (TPABr) was used as the template and sodium hydroxide (NaOH) as the base. The molar composition of the mixture was SiO_2_:TiO_2_:TPABr:H_2_O:NaOH = 1:0.035:0.10:30:x. The x varied from 0.02 to 0.07. The raw materials were thoroughly mixed by stirring. Before the mixture was transferred into a tumbled autoclave, the pH value was tested at room temperature by using a Mettler Toledo FiveEasy Plus FE28 pH acidometer (Zurich, Switzerland). Then, the mixture in the autoclave was treated at 443 K for 2 days. After cooling to room temperature, the pH value of the mixture was tested again. Then, the solid was obtained using filtration or centrifugation, dried, and calcined at 550 °C for 6 h. The small-sized zeolite (denoted as TS-1-small) was synthesized by using the same procedure with x = 0.03 except for the addition of 1.0 wt.% silicalite-1 zeolite in seed form to the starting mixture. The solids obtained were calcined at 823 K for 6 h, designated as TS-1-a, TS-1-b, TS-1-c, and TS-1-d with x values of 0.02, 0.03, 0.05, and 0.07, respectively.

### 2.3. Characterization

X-ray diffraction (XRD) measurements were performed on a Bruker Powder D8 Advance diffractometer (Billerica, MA, USA) at 40 kV and 40 mA using CuKa radiation (λ = 1.5418 Angstrom). DRUV/Vis spectra were recorded on a Shimadzu UV-2450 spectrophotometer (Kyoto, Japan) at 298 K using BaSO_4_ as a reference. FTIR spectra were recorded as KBr pellets on a Shimadzu IRPrestige-21 spectrometer (Kyoto, Japan). FTIR spectrum of pyridine adsorbed on TS-1 zeolites was tested at 298 K in a sealed reaction cell with KBr windows. A self-supporting disc (20 mg with 2.0 cm diameter) was prepared and evacuated at 723 K for 1 h. Subsequently, pyridine vapor was introduced and kept for 30 min. Excess pyridine was removed by vacuuming at 423 K for 30 min. The FTIR spectrum was collected in absorbance mode after the disc cooled to room temperature. Nitrogen adsorption–desorption isotherms were measured on a TriStar II 3020 sorption analyzer (Norcross, GA, USA) at 77 K. Elemental analyses (Si, Ti, and Na) were performed on an inductively coupled plasma optical emission spectrometer (Shimadzu ICPE-9000 spectrometer, Kyoto, Japan). Scanning electron microscopy (SEM) images were obtained on a JEOL JSM-7600F microscope (Tokyo, Japan) operated at 20 kV. Particle sizes were measured on a Malvern Zetasizer Nano ZS90 analyzer (Malvern, UK).

### 2.4. Catalytic Reaction

The oxidation reactions were carried with catalyst (25 mg), 1-hexene (5 mmol), and H_2_O_2_ (5 mmol) in methanol (5 mL) in a 20 mL glass reactor with 60 °C oil bath with stirring for 2 h. After reaction, the mixture was analyzed using Shimadzu GC-2014 gas chromatography (Kyoto, Japan) with a 30 m TC-1 capillary column and a flame ionization detector. Internal standards of cyclohexanone were used to calculate the mole of the reactants and epoxides. The H_2_O_2_ was determined with Ce(SO_4_)_2_ solution (0.1 M). The conversion and selectivity of epoxide (S_Epoxide_) and efficiency of H_2_O_2_ (S_H2O2_) were calculated by the following equations:

Conversion (%) = [(1-hexene)_i_ − (1-hexene)_f_]/(1-hexene)_i_ × 100

S_Epoxide_ (%) = (epoxy)_f_/[(1-hexene)_i_ − (1-hexene)_f_] × 100

S_H2O2_ (%) = [(1-hexene)_i_ − (1-hexene)_f_]/[(H_2_O_2_)_i_ − (H_2_O_2_)_f_] × 100

i and f represent initial and final molar value, respectively.

## 3. Results and Discussion

The optimal synthetic system was composed of tetrapropylammonium bromide (TPABr) as the structure-directing agent, colloidal silica as the silicon source, titanium tetra-n-butoxide as the titanium source, and NaOH as the pH-adjusting agent.

The active TS-1 zeolites were synthesized at a pH value range of 8.9 to 10.2, as shown in Table 1. With the increase in pH value, the sodium content in TS-1 products increased; meanwhile, the titanium content decreased. This implied the competitive relationship of Na^+^ and Ti^+^ cations to incorporate into the TS-1 product. More sodium in TS-1 would lead to a decrease in cooperated Ti content, which was consistent with previous reports [44,45]. The lower pH value after treatment in autoclave at 170 °C was mainly attributed to the decomposition of TPABr templates. The slight difference in TS-1-a was due to some amorphous raw materials in the TS-1-a sample, as indicated by the lower BET surface area and smaller microporous volume.

**Figure 1:**
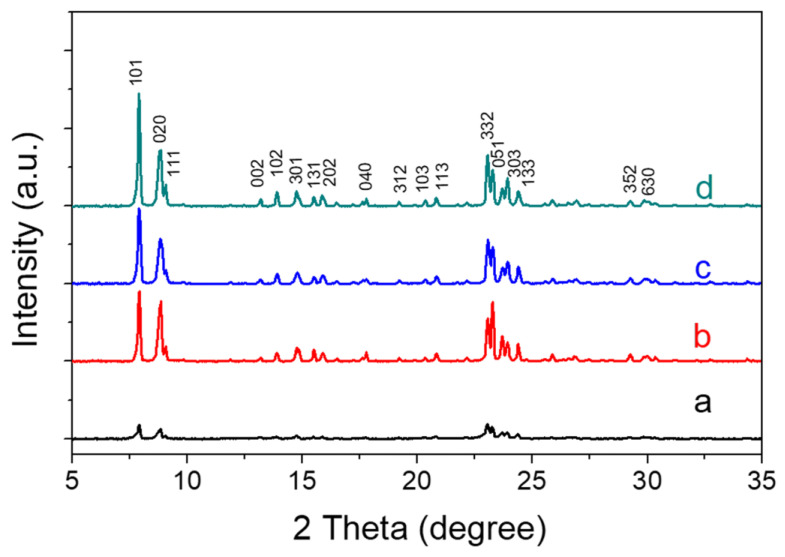
XRD patterns of TS-1 zeolites synthesized at different pH values. (**a**) TS-1-a, (**b**) TS-1-b, (**c**) TS-1-c, and (**d**) TS-1-d.

XRD patterns (Figure 1) showed that all the samples synthesized at different pH values had a pure MFI structure, but the TS-1-a sample showed weak peaks. This suggests that TS-1-a did not completely crystallize at the low pH value of 8.9, which was supported by the lower BET surface area and smaller microporous volume obtained from nitrogen adsorption characterization.

**Figure 2:**
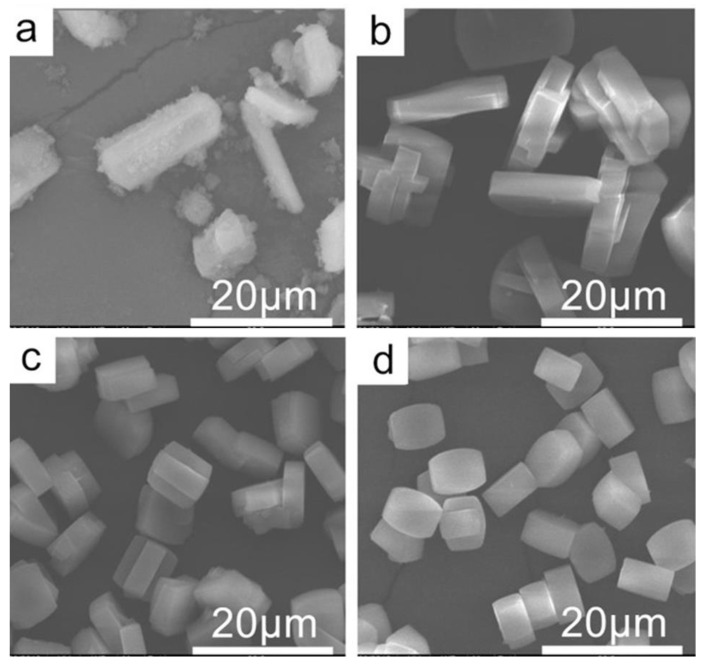
SEM images of TS-1 zeolites synthesized at different pH values. (**a**) TS-1-a, (**b**) TS-1-b, (**c**) TS-1-c, and (**d**) TS-1-d.

The SEM images of samples synthesized at different pH values are shown in Figure 2. They revealed the TS-1-a sample was composed of large crystals and amorphous small particles, confirming incomplete crystallization at lower pH values. This is also consistent with abovementioned results of lower microporous volume in nitrogen adsorption characterization and lower intensity in the XRD pattern. When the pH value was higher than 9.3, no amorphous particles were observed, indicating that all the raw materials became crystalline TS-1 zeolites. And, with the increase in pH value, it was found that the particle size of the TS-1 zeolites decreased gradually. It should be noted that the formation of zeolite crystals requires a certain alkalinity. There is a requirement for minimum alkalinity. The minimum alkalinity for complete crystallization is 9.3 pH. Considering this, as alkalinity increases, sodium ions increase, and the optimal alkalinity is 9.3 pH value after crystallization.

For catalytic applications of TS-1 zeolites, it has been proven that only tetrahedral Ti species, namely, framework Ti species, provide catalytic activity [20,30,45]. Other species, including extra-framework Ti species and anatase-like TiO_2_ particles, do not contribute to catalytic performance.

**Figure 3:**
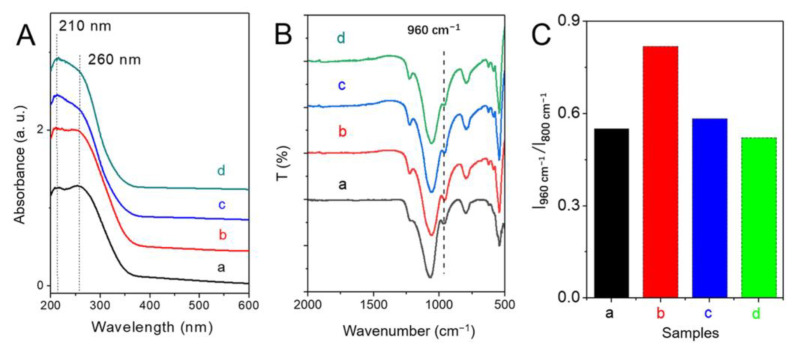
(**A**) DRUV-visible spectra, (**B**) FTIR spectra, and (**C**) intensity ratio of 960 cm^−1^ to 800 cm^−1^ in FTIR spectra of TS-1 zeolites synthesized at different pH value. (a) TS-1-a, (b) TS-1-b, (c) TS-1-c, and (d) TS-1-d.

Diffuse reflectance UV-visible (DRUV-visible) and FTIR spectroscopy are powerful techniques for detecting the coordination states of Ti species in TS-1. The absorbance peak at approximately 210 nm in the DRUV-visible spectrum and the band at 960 cm^−1^ in FTIR have been widely accepted as proof of the presence of framework Ti species [20,35,46]. As shown in Figure 3A, all the samples showed 210 nm and 260 nm bands, indicating the presence of both framework Ti species and extra-framework Ti species. No obvious adsorption band at about 330 nm was detected, implying no anatase-like TiO_2_ particles present in these samples. FTIR spectra (Figure 3B,C) showed that all the samples had peaks at 960 cm^−1^, which is attributed to a stretching vibration mode of [SiO_4_] perturbed by adjacent framework Ti species [46,47]. Importantly, the band intensity at 960 cm^−1^ proportionally increases if the amount of the framework Ti in TS-1 increases. As shown in Figure 3C, TS-1-b displayed relatively higher intensity in this characteristic band, suggesting higher framework Ti species content in this sample.

To evaluate the catalytic performance of the prepared TS-1 zeolitic catalysts, epoxidation reactions of 1-hexene with hydrogen peroxide as oxidant were performed. As shown in Table 2, two samples from traditional synthesis methods were used as comparative samples. TS-1-co with 100–500 nm particle size was purchased from the Catalysis Society of Japan. Sample TS-1-Na0.02 with 100–200 nm crystal size was synthesized with 0.02 of Na/Si molar ratio in the traditional synthetic system with a pH of about 12. TS-1-Na0.02 showed very low catalytic activity for 1-hexene epoxidation, indicating the great influence of Na^+^ ions on the synthetic system. At such high alkalinity, the presence of sodium ions affects the entry of titanium into the skeleton, as indicated by the higher Si/Ti ratio. Therefore, the content of alkali metal cations should be strictly controlled in the traditional synthetic processes.

After decreasing pH value, the content of titanium greatly increased compared to TS-1-Na0.02 synthesized using the traditional system. At the same time, the content of sodium ions in the sample also increased. From the catalytic results, the TS-1-a sample without total crystallization achieved a 7.8% conversion of 1-hexene. TS-1-b showed the best conversion of 9.3% among four synthesized samples. Although the crystal sizes of TS-1-c and TS-1-d samples were smaller than that of TS-1-b (Figure 2), they showed lower catalytic activity because of their lower titanium content and slightly higher content of Na^+^ (Table 1). Therefore, both the titanium content and sodium content in TS-1 zeolite can affect catalytic activity. The crucial factor in synthesizing the active TS-1 catalyst was increasing the content of titanium and decreasing the content of Na cations in the final samples. As mentioned above in Table 1, the titanium content in TS-1 products would decrease with the increase in pH value. The sodium content would increase with the increase in pH value. So, the key to solving the problem of the adverse effects of Na^+^ ions during the synthesis processes was to adjust precisely the pH value to as low as possible under the premise that the raw materials could crystallize to form crystals.

It is excited that, for the first time, active TS-1 zeolites can be synthesized by using common and cheap NaOH as a base. This is a significant step towards the extremely low-cost synthesis of this important commercial catalyst. However, the catalytic activity was still lower due to the large crystal size. A sample of TS-1-b with 9.3% 1-hexene conversion had a crystal size of 15–18 μm in length and 4–5 μm in thickness, which was much larger than the sub-micrometer-sized TS-1 crystal size (for example, 100–500 nm for TS-1-co and 100–200 nm for TS-1-Na0.02) synthesized using traditional synthesis systems. A smaller crystal size is beneficial for the utilization of micropores inside zeolites and improves catalytic performance. For TS-1 catalysts, it was reported that reducing the size of zeolite crystal can improve its catalytic activity [30].

**Figure 4:**
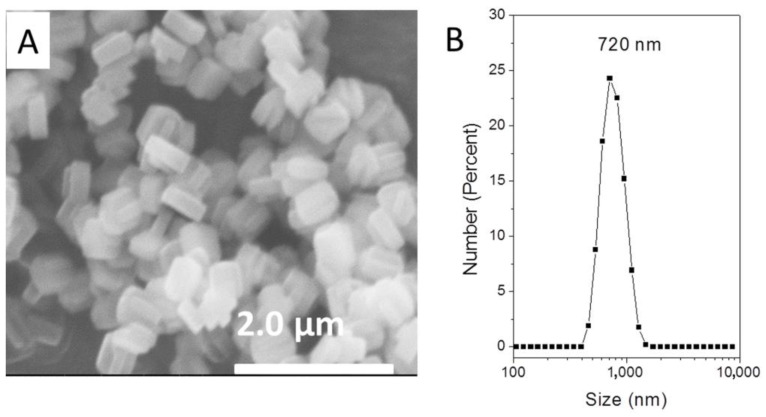
(**A**) SEM image and (**B**) particle size distribution of TS-1-small sample with sub-micrometer crystals.

Although increasing alkalinity allowed for synthesizing TS-1 zeolites with smaller particle sizes, the TS-1 zeolites prepared at high alkalinity showed low catalytic activity using NaOH as the base for adjusting alkalinity, as discussed above. In order to reduce crystal size, a method of adding nanosized silicalite-1 zeolites as seeds was employed based on reports from a previous paper (detailed synthesis in the Setion 2). As shown in Figure 4, the SEM image indicated that the particle sizes were much smaller than those synthesized without adding seeds. The particle size distribution (Figure 4B) indicated that the mean size of the TS-1 crystals was 720 nm.

**Figure 5:**
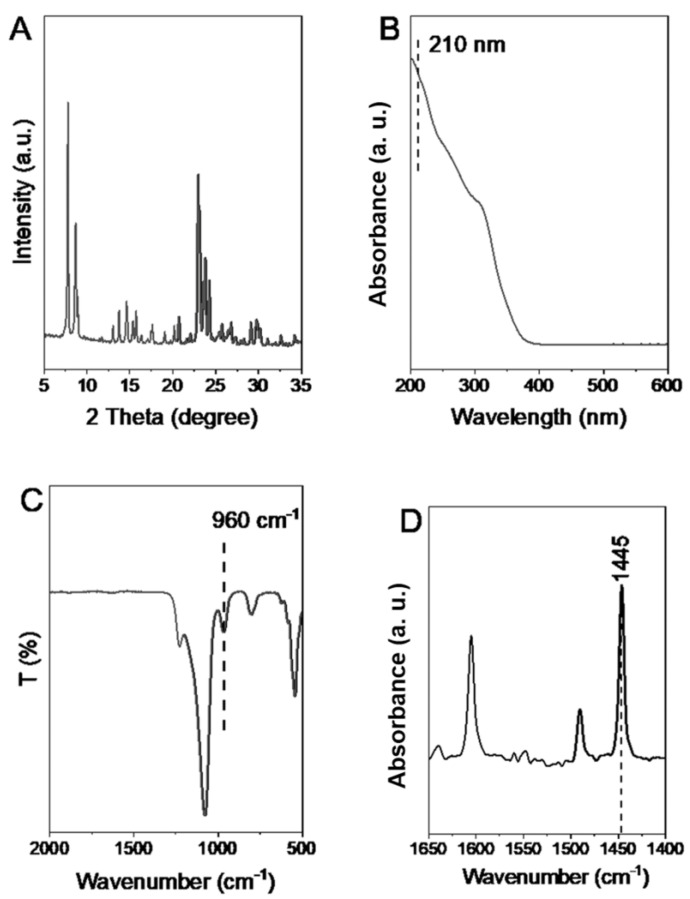
(**A**) XRD pattern, (**B**) DRUV-visible spectrum, (**C**) FTIR spectrum, and (**D**) FTIR spectra of pyridine adsorbed on zeolites for TS-1-small sample with sub-micrometer crystals.

The XRD pattern showed that TS-1-small had a crystalline MFI structure (Figure 5A). The presence of active framework titanium was detected by the observation of a 210 nm band in the DRUV-visible spectrum (Figure 5B) and a peak of 960 cm^−1^ in the FTIR spectrum (Figure 5C). A band at 1445 cm^−1^ associated with pyridine adsorbed on Lewis acid sites was observed in the FTIR spectrum of pyridine characterization (Figure 5D), indicating the Lewis acid characteristic of TS-1-small zeolites. In addition, the BET surface area and the microporous volume of TS-1-small was 401 m^2^ g^−1^ and 0.17 cm^3^ g^−1^, respectively.

The catalytic performance of TS-1-small with 720 nm sized crystals is shown in Table 2. It achieved a similar conversion and a higher epoxide selectivity compared to commercial TS-1 catalyst during catalytic oxidation of 1-hexene in the presence of H_2_O_2_, indicating great potential for commercialization in consideration of its competitive price (Table S1 in the Supplementary Materials) and environmentally benign issues.

## 4. Conclusions

In summary, for synthesizing Lewis acid zeolites, especially Ti-containing zeolites, the amount of Na^+^ in raw material is highly restricted in the traditional process because trace amounts greatly decrease the catalytic activity of final zeolites. In this paper, we reported a method for synthesizing low-cost and high-performance TS-1 zeolites by directly using sodium hydroxide (NaOH) as the base. The key was solving the problem posed by the adverse effects of Na^+^ ions during synthesis processes via precisely adjusting the pH value to as low as possible under the premise that the raw materials would crystallize to form crystals. TS-1 zeolites with 9.3% 1-hexene conversion were directly synthesized under a pH value of about 9.3. Furthermore, assisted with nanoseeds, sub-micrometer-sized TS-1 zeolites with 23.0% 1-hexene conversion and 95% epoxide selectivity were obtained, showing similar catalytic performance to current commercial TS-1 zeolites, indicating great commercial potential. Moreover, the present report opens the possibility of preparing other high-performance Lewis acid zeolites (e.g., Sn, Zr, etc.) by using cheap, stable, and nonvolatile inorganic alkalis.

## Figures and Tables

**Table 1:** Composition and porosity of final TS-1 zeolites under different synthetic pH values.

Sample	pH Value ^1^	pH Value ^2^	Si/Ti (mol/mol)	Na (wt.%)	S_BET_ ^3^ /m^2^ g^−1^	V_Micro._ ^4^ /cm^3^ g^−1^
TS-1-a	8.9	8.4	25	0.412	204.5	0.07
TS-1-b	9.3	7.6	31	0.392	388.4	0.17
TS-1-c	9.4	7.0	35	0.560	393.9	0.17
TS-1-d	10.2	7.8	45	0.567	385.3	0.16

^1^ pH value before treatment in autoclave at 170 °C. ^2^ pH value after treatment in autoclave at 170 °C. ^3^ Specific surface area from Brunauer–Emmett–Teller (BET) method. ^4^ Microporous volume and external specific surface area evaluated using t-plot curves.

**Table 2:** Catalytic oxidation of 1-hexene with H_2_O_2_.

Sample	Si/Ti (mol/mol)	Na (wt.%)	Conversion (%)	S_Epoxide_ ^1^ (%)	S_H2O2_ ^2^ (%)
TS-1-a	25	0.412	7.8	99.0	63
TS-1-b	31	0.392	9.3	98.7	58
TS-1-c	35	0.560	4.4	99.0	47
TS-1-d	45	0.567	3.0	99.0	53
TS-1-small ^3^	35	0.278	23.0	95.0	74
TS-1-co ^4^	45	-	24.4	90.8	80
TS-1-Na0.02 ^5^	71	0.180	1.2	77.0	99

Reaction condition: catalyst (25 mg), methanol (5 mL), 1-hexene (5 mmol), H_2_O_2_ (5 mmol), temperature 60 °C, 2 h. ^1^ Selectivity of epoxide. ^2^ Efficiency of H_2_O_2_ towards the oxidation of 1-hexene. ^3^ TS-1 samples with 720 nm crystal size. ^4^ TS-1-co from Catalysis Society of Japan. ^5^ TS-1-Na0.02 was synthesized in the presence of Na^+^ ions (0.02 of Na/Si molar ratio) in the traditional synthetic system with pH about 12 [45].

## Data Availability

Data can be shared upon request to the authors.

## Supplementary Materials

The following supporting information can be downloaded at: https://www.mdpi.com/article/10.3390/nano13162296/s1, Table S1. A comparison of this method with the traditional synthesis processes.
