# Supplementary material for: Approaching High-Performance TS-1 Zeolites in the Presence of Alkali Metal Ions via Combination of Adjusting pH Value and Modulating Crystal Size
Source: Nanomaterials (Basel). 2023 Aug 10;13(16):2296. doi: 10.3390/nano13162296 (PMC10458067; doi:10.3390/nano13162296)
Supplement: Supplementary file 1 [file nanomaterials-13-02296-s001.zip › nanomaterials-2511511-supplementary.pdf]

Supporting Information for

Approaching high-performance TS-1 zeolites in the presence of alkali metal ions via combination of adjusting pH value and modulating crystal size

Geng Li [1, †], Kairui Fu [1,2, †], Fulin Xu [1], Tianduo Li [1,2], Yunan Wang [3,*] and Jingui Wang [1,3,*]

[1] Shandong Provincial Key Laboratory of Fine Chemicals, School of Chemistry and Chemical Engineering, Qilu University of Technology (Shandong Academy of Sciences), Jinan 250353, China; iamli-geng97@163.com (K. F.), kr475686761@163.com (G.L.), xufulin97@163.com (F.X.), ylpt6296@vip.163.com (T. L.), JGWang@qlu.edu.cn (J. W.)

[2] School of Chemistry and Chemical Engineering, University of Jinan, Jinan 250022, China; kr475686761@163.com (K. F.), ylpt6296@vip.163.com (T. L.)

[3] Key Laboratory of Advanced Fuel Cells and Electrolyzers Technology of Zhejiang Province, Ningbo In-stitute of Materials Technology and Engineering, Chinese Academy of Sciences, Ningbo 315201, China; wangyunan@nimte.ac.cn (Y. W.), JGWang@qlu.edu.cn (J. W.)

[†] These authors contributed equally to this work.

[*] Correspondence: JGWang@qlu.edu.cn (J. W.), wangyunan@nimte.ac.cn (Y. W.)

Table S1. A comparison of this method with the traditional synthesis processes.

Method	Temperature [1] (°C)	Time [2] (Day)	Yield [3] (%)	Total Cost [4] (CNY/kg)	SiO_2 Cost (CNY/kg)	Template Cost (CNY/kg)	Other Costs (CNY/kg)
Our method	170	2	90	100	35	60	5
Traditional [5]	175	10	86	500	88	408	4

[1] Crystallization temperature in autoclave. [2] Crystallization time in autoclave. [3] The mass of solid product based on the amounts of SiO_2 and TiO_2 added in raw materials. [4] Tentative calculation based on raw material costs, excluding energy, equipment, and other costs. Our method, colloidal silica (30%) with price of 10 CNY/kg as SiO_2 raw material and TPABr (94%) with price of 120 CNY/kg as template, 3.5 kg of 30 % colloidal silica and 0.5 kg 94% TPABr are required for each kilogram of catalyst product. For traditional method, TEOS (96%) with price of 22 CNY/kg as SiO_2 raw material and 25% TPAOH with price of 60 CNY/kg as template, 4.0 kg of 96 % TEOS and 6.8 kg 25% TPAOH are required for each kilogram of catalyst product. Other costs include the costs of TiO_2 raw materials, NaOH, water, etc. [5] The traditional synthetic system referred to patent (M. Taramasso, G. Perego, B. Notari, 1983, US Pat. 4,410,501).
